# Conductive cellulosic pulp composites reinforced with copper-silica hybrid

**DOI:** 10.1038/s41598-025-17701-y

**Published:** 2025-09-12

**Authors:** Hoda Sabry Othman, Azza Abbas M. Ward, Sawsan Dacrory, Galal A. M. Nawwar

**Affiliations:** 1 Green Chemistry Department, National Research Centre, Giza, Egypt; 2 Microwave Physics and Dielectrics Department, National Research Centre, Giza, Egypt; 3 Cellulose and Paper Department, National Research Centre, Giza, Egypt

**Keywords:** Sustainability, Rice straw, Black liquor, Lignin/silica hybrid, Dielectric, Conductivity, Chemistry, Engineering, Environmental sciences, Materials science

## Abstract

The rice straw pulp black liquor (RSBL) is high-alkalinity wastewater rich in silica, which renders its known use as a fuel in pulping mills difficult. We explored the economic utilization of RSBL *via *alkaline precipitation, affording metal lignin/silica hybrids. Our current study focuses on exploring the electrical characteristics of newly developed composites by integrating different ratios of a prepared conductive copper/lignin/silica (Cu–LSF) hybrid into rice straw cellulosic pulp, produced through solar pulping. We also compare the properties of these rice straw-based composites with those prepared using conventionally pulped bagasse. All the prepared samples were characterized using a combination of techniques, including X-ray diffraction, and scanning electron microscopy. Their permittivity and dielectric loss were also measured. Rice straw-based composites containing higher dielectric silica contents exhibited superior permittivity and dielectric loss performance compared to bagasse-based composites.

## Introduction

Dielectric materials are highly sought after for their use in electronics and devices^1^. They offer a winning combination of insulating properties, excellent mechanical strength, light weight, ease of processing, low production cost, and superior breakdown strength. This makes them ideal for components like capacitors, where they play a crucial role in energy storage^2,3^. However, a significant environmental concern arises from the widespread use of non-degradable, low-durability synthetic polymers in many popular electronic gadgets^4,5^.

Cellulose, an abundant natural resource, is gaining attraction as a versatile material due to its biodegradability, renewability, and global availability. This makes it an attractive alternative to conventional materials in a wide range of applications^6–12^. In the realm of electronics, finding sustainable options is essential. Cellulose offers a crucial solution for conductive applications due to its environmentally friendly nature, abundance, and lightweight properties^13,14^. It helps mitigate environmental concerns, diversifies electronic product options, and elevates the value of this low-cost material, drawing considerable interest from researchers and manufacturers alike.

The inherent dielectric properties of cellulose, long utilized in various applications, are now sparking renewed interest in its nanofibrillated film form for flexible electronics^15,16^. Flexible conductive composites (FCCs), made by dispersing conductive materials within an elastomer, offer advantages including large reversible deformation, rapid response, affordability, and ease of processing, making them highly sought after^17^.

The growing demand for flexible electronic devices has created a critical need for polymer materials that simultaneously exhibit high breakdown strength, a low loss tangent, and excellent electrical conductivity. FCCs are particularly appealing for electronic applications due to their ability to maintain electrical conductivity even when mechanically deformed^18,19^.

The unique characteristics of nanomaterials—particularly their greater surface area and excellent mass, heat, and charge transfer capabilities—make them essential for enhancing energy conversion and storage applications^20,21^.

By integrating conductive materials into the cellulose 3D structure, naturally insulating cellulose could be made conductive. This conductive cellulose opens up exciting possibilities for building flexible and portable electronic devices^21–23^. Imagine everything from simple electronic components to complex integrated systems like Organic Light-Emitting Diodes (OLEDs), Solar Cells, Supercapacitors (SCs), Electromagnetic Shields, Lithium Batteries, Sensors, and Electrodes. Hassan et al.^15^ have successfully isolated nanofibrillated cellulose (NFC) and combined it with polyvinylpyrrolidone (PVP) and silver nanoparticles. This combination creates flexible films that show promising mechanical and electrical properties, making them suitable for use as antistatic and electrostatic dissipative materials.

For a long time, disposing of black liquor byproduct from pulping of rice straw has been an environmental burden and posed a significant disposal challenge for the industry. Coagulation is regarded as one of the most cost-effective methods for treating diverse organic wastewater. The removal of chemical oxygen demand (COD) and color from paper mill effluent using a coagulation process was studied. Various coagulants, including aluminum chloride, poly aluminum chloride, and copper sulfate, were tested. With copper sulfate as the coagulant, optimal conditions of pH 6 and a mass loading of 5 g/L yielded significant reductions: 76% for COD and 78% for color^24^.

Aiming clean economic production of rice straw pulp, we successfully precipitated nano-sized metal lignin-silica-fatty acids complexes by treating the black liquor obtained as a co-product from the rice straw pulping process with metal salts^25–29^. We thoroughly examined the incorporation of these metal complexes as functional additives into various rubber matrices as well as in coating formulations. The investigating morphological, physical, mechanical, and electrical properties recommended the utilization of such biomass-derived complexes as valuable green additives for several industrial applications^25–29^.

As copper displays the highest conductivity of any non-precious metal^30^, accompanied by its good resistance to corrosion and ease of joining, it is regarded as an ideal electrical conductor for applications like cables, transformers, and motor windings. Copper lignin-silica-fatty acids (Cu–LSF) hybrid, precipitated from rice straw black liquor *via* coagulation by copper sulfate^25^, demonstrated promising mechanical and conductive properties when incorporated into NBR and EPDM rubber matrices^25–27^.

**Fig. 1:**
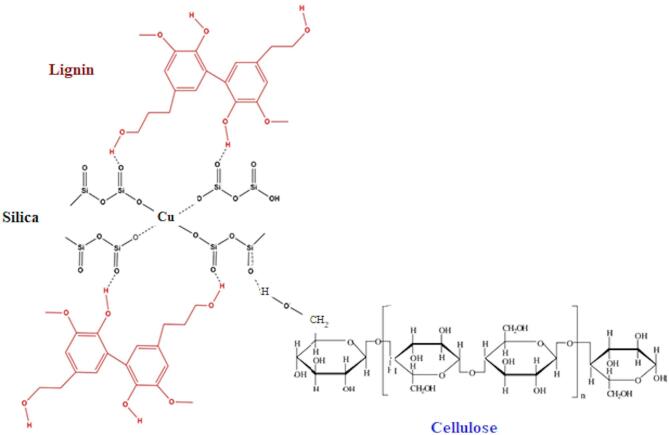
Cu–LSF complex integrated within the cellulosic pulp.

In the current study, we aim to investigate the conductive properties of the resultant composites from incorporating the Cu–LSF hybrid into the cellulosic pulp produced from the rice straw solar pulping process^28^ (Fig. 1). Such environmentally safe, zero-waste local technology, which repurposes both rice straw pulp and black liquor for various applications^31–38^, deserves attention and widespread adoption due to its potential for sustainable material cycles and waste minimization. It’s particularly beneficial for countries grappling with non-wood agricultural waste. Therefore, this work examines the dielectric and conductive characteristics of Cu–LSF-loaded rice straw pulp composites, drawing comparisons with bagasse-based composites.

## Materials and methods

### Materials

Rice straw was supplied from Beheira Governorate, Egypt, and then subjected to a mild solar-aided pulping process according to our previously reported method^28^ to obtain rice straw pulp. We bleached the resultant pulp as described in our previous work^39^. Bagasse raw material was supplied from the Quena Company of Paper Industry, Egypt. The chemical composition of the bagasse raw material was determined according to TAPPI standards (Klason lignin, Pentosan, alpha cellulose, and ash content), and the results are 22, 44.4, 28.1, and 1.4, respectively.

### Synthesis of copper/lignin/silica (Cu–LSF) hybrid

The Cu–LSF hybrid was synthesized using black liquor obtained from rice straw pulping, according to our previously published methodology^25^.

### Preparation of Cu–LSF-based cellulosic blends

In a comparative study of solar-pulping-derived rice straw pulp and bagasse pulp, two sample series were prepared. Each sample consisted of 1 g pulp, 0.5 g carboxymethyl cellulose (CMC), 15 mL H_2_O, and different volumes of water-suspended Cu–LSF (10, 20, and 50 mL), as outlined in Table 1. The samples were allowed to stir overnight, then poured in plastic cups and put in the freeze dryer for 24 h. To improve crosslinking and water resistance, all the prepared mixtures were treated with FeCl_3_ solution, washed, and dried. It is worth noting that a third series of bagasse pulp-based composites was prepared without immersion in FeCl_3_ solution.

**Table 1:** Composition of rice straw pulp and Bagasse pulp-based composites.

	Pulp (gram)		CMC (gram)		H_2_0 (mL)	Cu–LSF solution (mL)
R1 (Blank)	Rice straw	1	0.5	Immersed in FeCl_3_ solution	15	–
R2		1	0.5		15	10
R3		1	0.5		15	20
R4		1	0.5		15	50
B1 (Blank)	Bagasse	1	0.5		15	–
B2		1	0.5		15	10
B3		1	0.5		15	20
B4		1	0.5		15	50
NF1 (Blank)	Bagasse	1	0.5	Without immersion in FeCl_3_ solution	15	--
NF2		1	0.5		15	5
NF3		1	0.5		15	10

### Characterizations

#### **X-ray diffraction (XRD)**

The XRD patterns of cellulose, CMC, Cu-LSF complex, R1, and R4 were investigated on a Diano X-ray diffractometer using CuKα radiation source energized at 45 kV and a Philips X-ray diffractometer (PW 1930 generator, PW 1820 goniometer) with a CuK radiation source (λ = 0.15418 nm), at a diffraction angle range of 2θ from 10 to 80° in reflection mode.

#### Scanning electron microscopy (SEM) and energy-dispersive X-ray spectroscopy (EDAX)

Scanning electron microscopy (SEM) using a JEOL JSM 6360LV (operated at 10–15 kV) attached to the EDAX unit was employed to examine the surface structure of the prepared samples. Prior to analysis, the samples were sputter-coated with a thin gold layer under carefully controlled deposition rate and target distance to improve conductivity and prevent damage.

#### Fourier-transform infrared (FT-IR) spectroscopy

FT-IR spectra were recorded using a JASCO FT/IR-4100 LE spectrometer (Easton, MD, USA) operating in absorption mode within the 4000–400 cm⁻¹ wavenumber range.

#### Dielectric spectroscopy technique

The dielectric and conductivity measurements were carried out by means of a high-resolution broadband impedance analyzer (Schlumberger Solartron 1260), an electrometer, an amplifier, and a measuring cell. The frequency range of the applied AC electric field was between 0.1 Hz and 1 MHz. Electromagnetic shielding minimized low-frequency noise. The measurements were automated by interfacing the impedance analyzer with a personal computer through a GPIB cable IEE488. Commercial interfacing and automation software, LabVIEW, was used for the acquisition of data. Prior to the sample measurements, the calibration was performed to eliminate the effect of stray capacitance. The error in ε’ and ε’’ amounts to 1% and 3%, respectively. The temperature of the samples was controlled by a temperature regulator with a Pt 100 sensor. The error in temperature measurements amounts to 0.5 °C. To avoid moisture, the samples were stored in desiccators in the presence of silica gel. Thereafter, the sample was transferred to the measuring cell and left with P_2_O_5_ until the measurements were carried out.

## Results and discussion

### X-ray diffraction (XRD) analysis

**Fig. 2:**
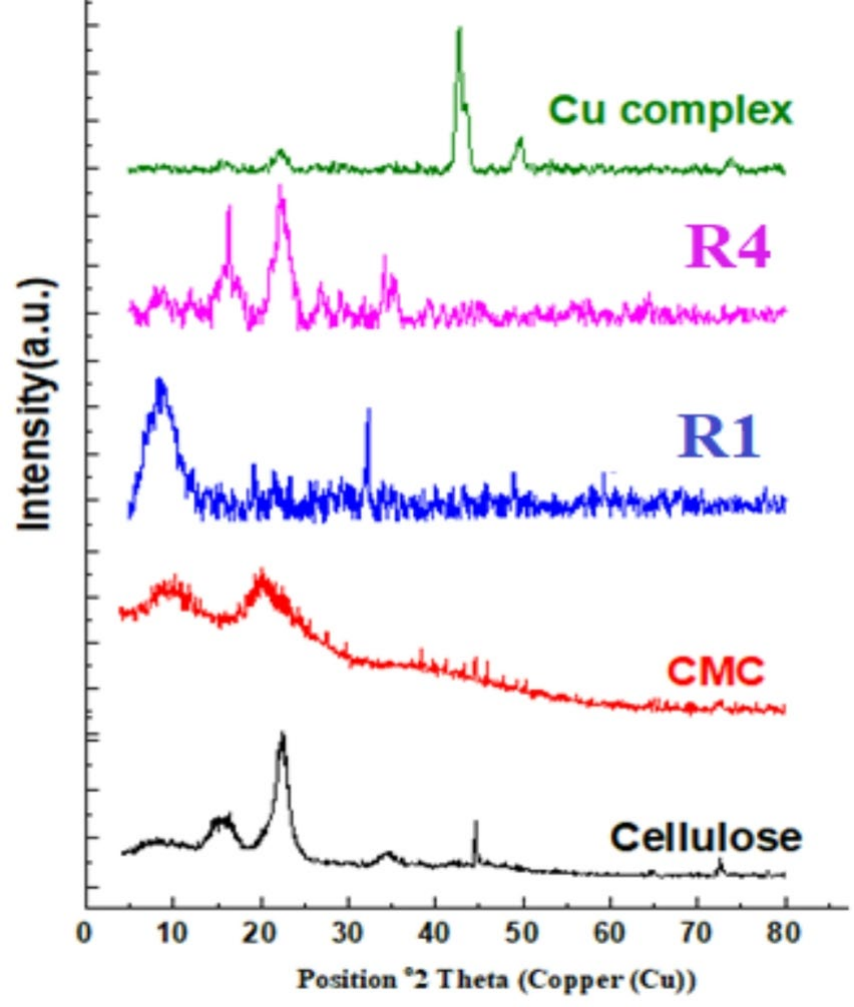
XRD of cellulose, CMC, Cu-LSF complex, R1 and R4.

As evidenced by Fig. 2, the XRD pattern of cellulose exhibits characteristic peaks at 2θ = 12° and 22°. Notably, the CMC pattern shows a broadening of these peaks, which is indicative of an expanded amorphous region. The XRD pattern of the Cu-LSF complex displays peaks at 2θ = 24°, assignable to silica, and at 44° and 50°, corresponding to copper. The weak copper-related peaks in the R1 and R4 patterns are likely due to the low concentration of copper in those composites.

### SEM and EDAX

**Fig. 3:**
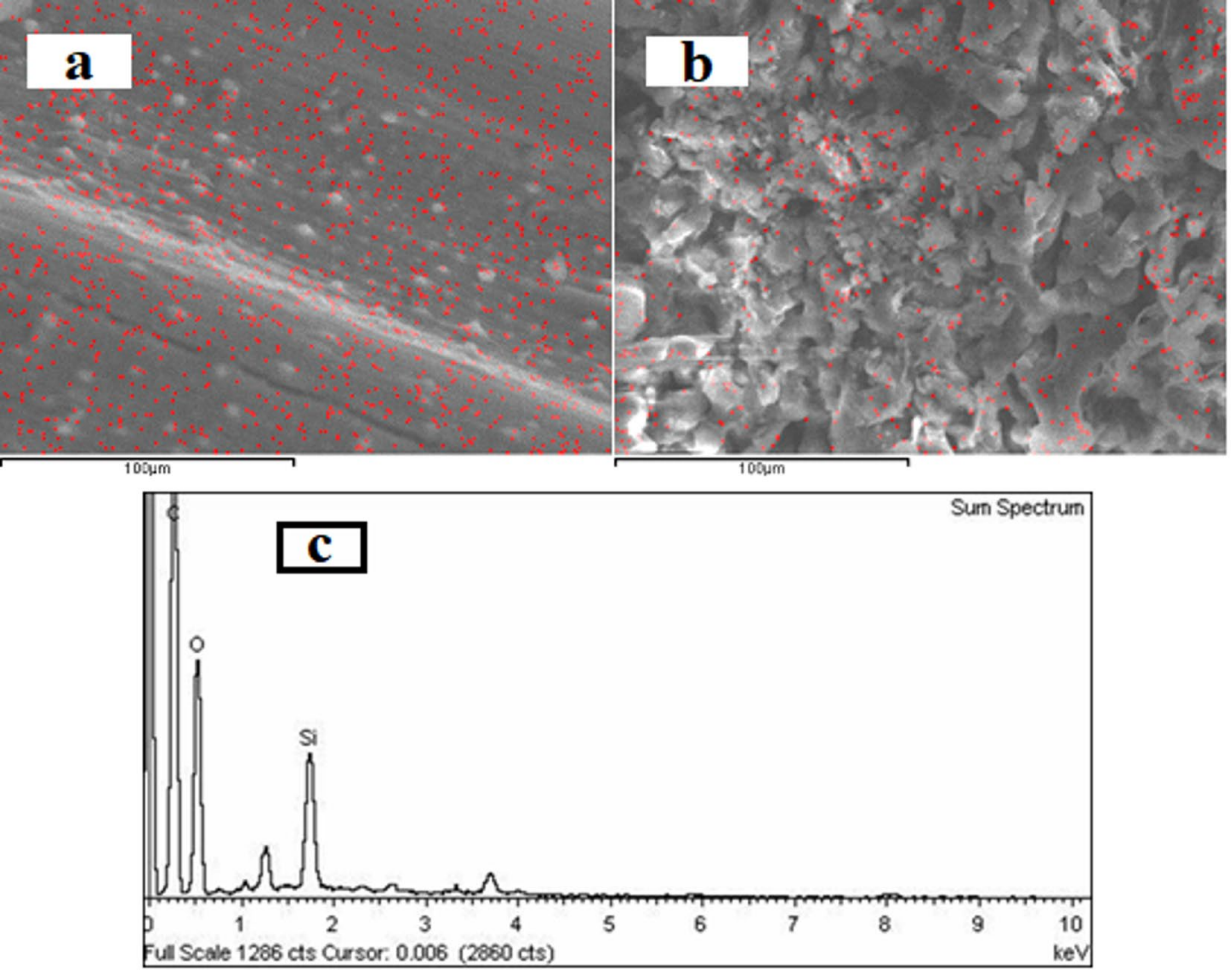
SEM for rice straw **a** raw material, and **b** solar-pulped material; **c** EDAX of the solar-pulped material.

SEM images in Fig. 3 illustrate the surface morphology of raw rice straw (Fig. 3a) and rice straw after alkaline solar pulping (Fig. 3b), with silica present on the surface of the raw rice straw (Fig. 3a) and reduced after pulping (Fig. 3b). The micrograph of the pulped material (Fig. 3b) reveals long fibers, suggesting good mechanical strength. Furthermore, EDAX of the pulped material (Fig. 3c) provides a graphical representation of the silica percentage in the solar-pulped rice straw fibers. This data suggests that a considerable amount of silica remains in the pulp, implying a lower concentration of silica in the black liquor generated by this method compared to conventional pulping.

**Fig. 4:**
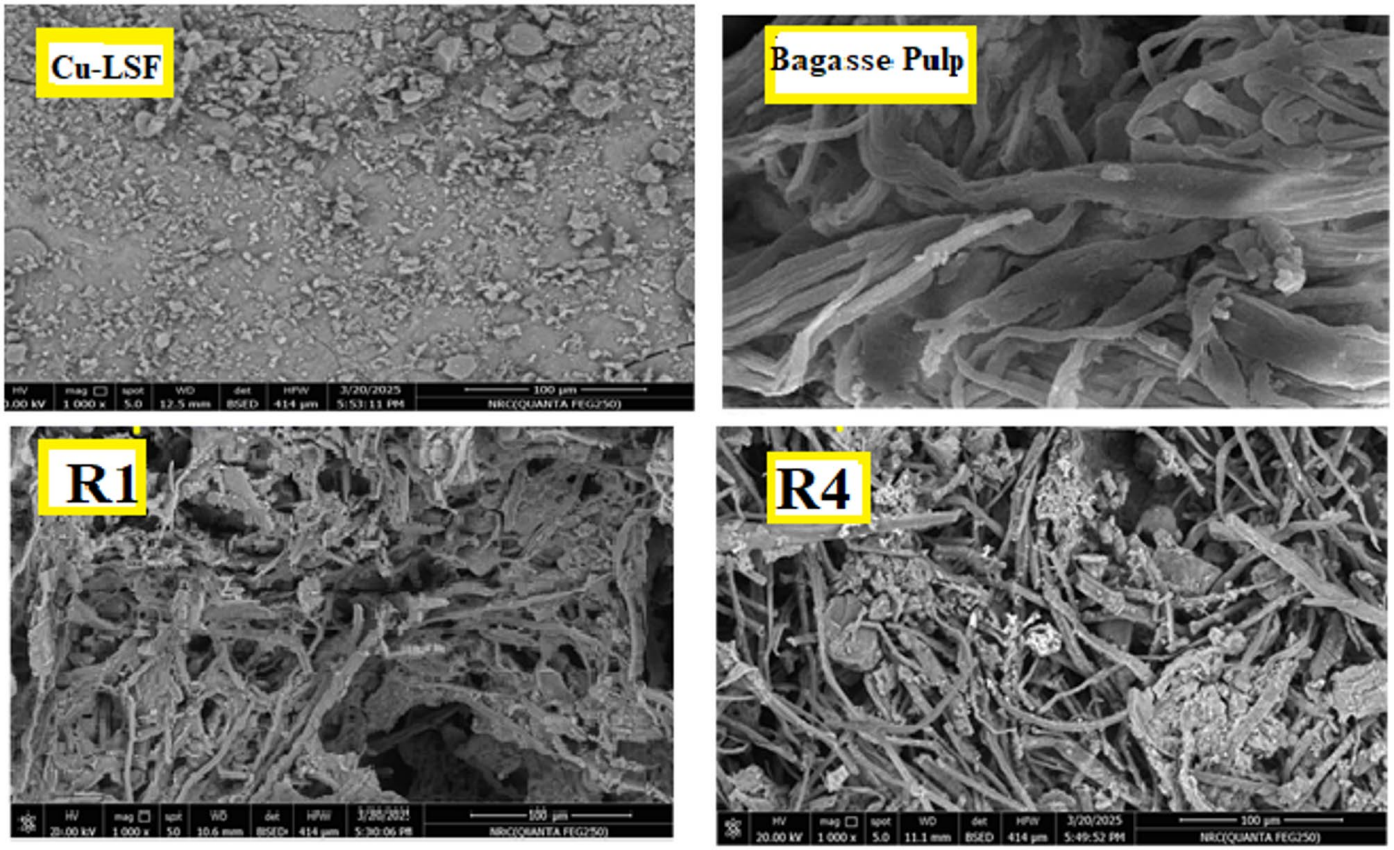
SEM of Cu–LSF complex, Bagasse pulp, R1, and R4.

It is worth noting that, a clear difference in pulp morphology: bagasse pulp (Fig. 4) lacked surface silica, likely due to sugarcane bagasse’s low silica content^40^, unlike rice straw pulp.

Moreover, Fig. 4 clearly illustrates the integration of the Cu–LSF (50 ml) within the rice straw cellulosic pulp matrix (R4), unlike the control sample (R1).

**Fig. 5:**
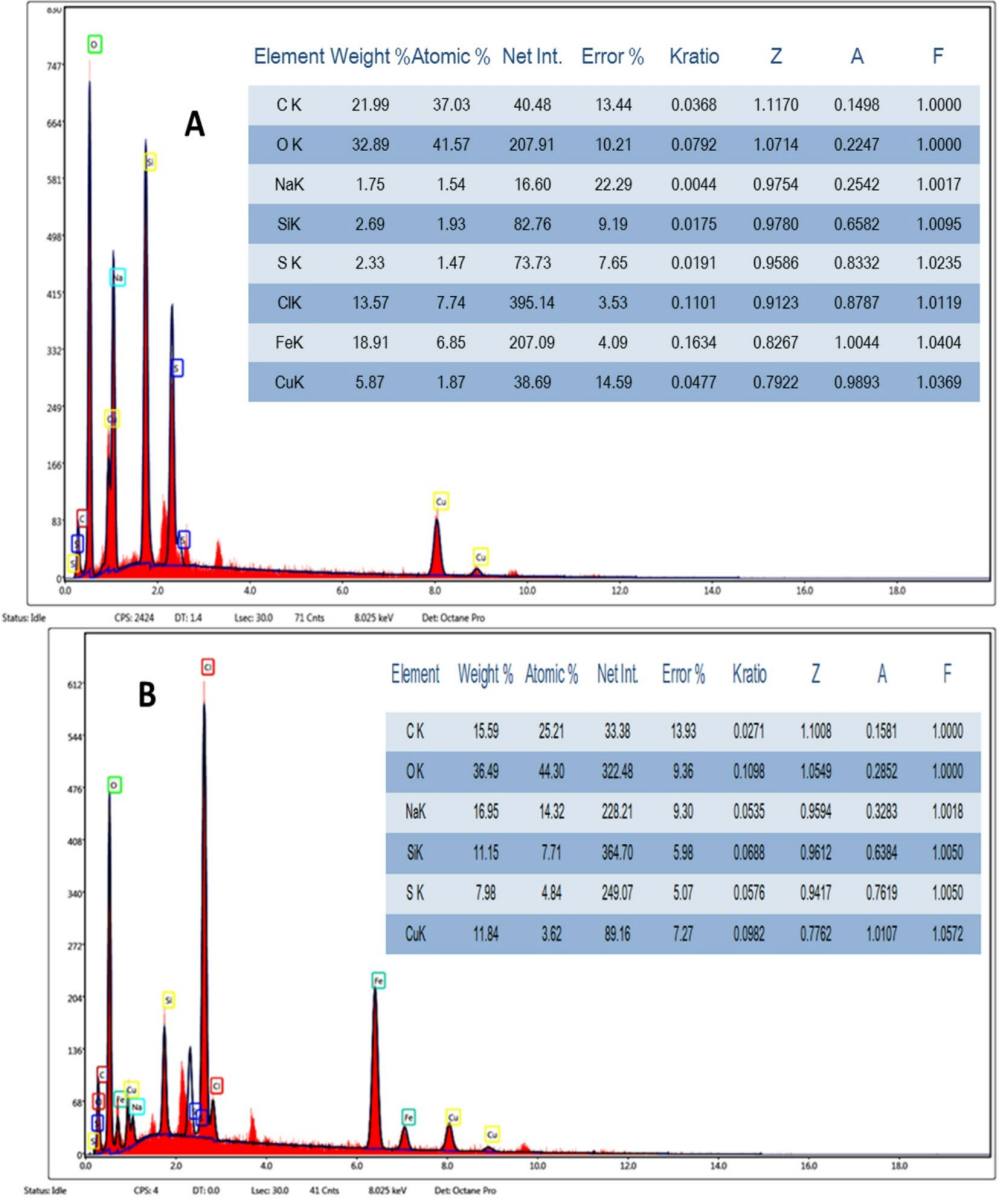
**A** EDAX of Cu–LSF complex, and **B** EDAX of R4 containing 50 ml of Cu–LSF complex.

EDAX analysis of Cu–LSF (Fig. 5a) confirmed the presence of expected elements like copper, carbon, silicon, and sodium. These elements were also detected in the R4 pulp matrix after Cu–LSF incorporation, as shown in Fig. 5b. This confirms successful incorporation of Cu–LSF into the pulp matrix, which may contribute to enhanced dielectric behavior.

### FT-IR spectroscopy

**Fig. 6:**
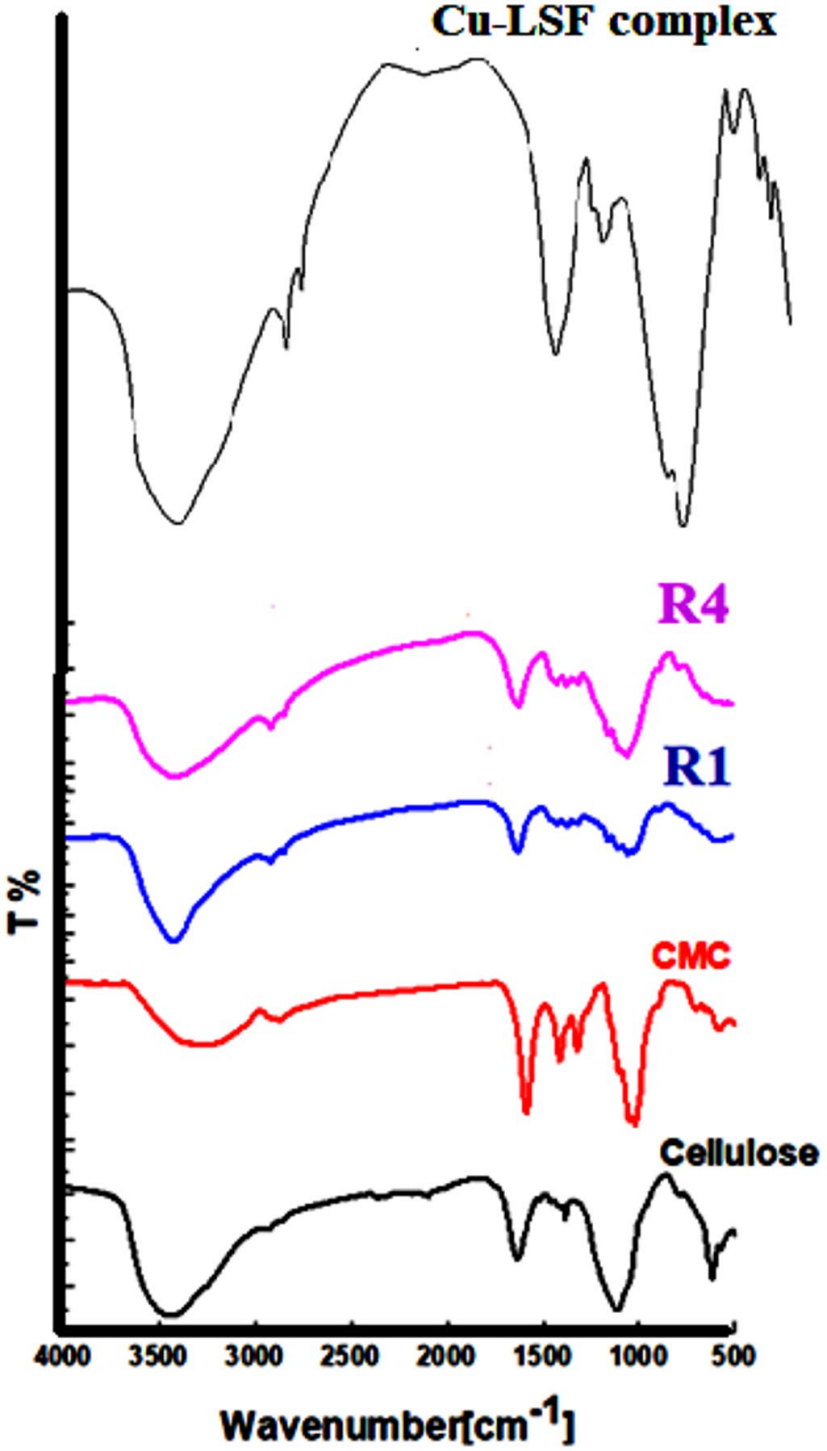
FTIR of cellulose, CMC, control (R1), R4 composite, and Cu–LSF complex.

Figure 6 presents FTIR analysis of cellulose, carboxymethyl cellulose (CMC), Cu-LSF complex, a control rice straw pulp (R1), and a composite (R4) containing 50 mL of Cu–LSF complex solution. The spectrum for cellulose exhibits characteristic peaks for OH stretching (3500–3000 cm⁻¹), CH stretching (2900 cm⁻¹), absorbed water (1650 cm⁻¹), and C-O-C ether linkages (1100 cm⁻¹). CMC displays similar peaks, with variations at 1596, 1400 cm⁻¹, and 1200 cm⁻¹ attributed to C=O (hydrogen bonding), CH₂, and C–H bond vibrations, as well as C–H asymmetric bending^41^.

The presence of the Cu–LSF complex’s characteristic peaks^25^, including bands for the lignin (OH at 3450 cm⁻¹; aromatic at 3200 cm⁻¹; and aliphatic at 2850 cm⁻¹), and the carbonyl absorption band (1650 cm⁻¹), is indicated with low intensity in the R4 composite, which is likely due to the partial interaction with the pulp matrix.

### Dielectric spectroscopy technique

**Fig. 7:**
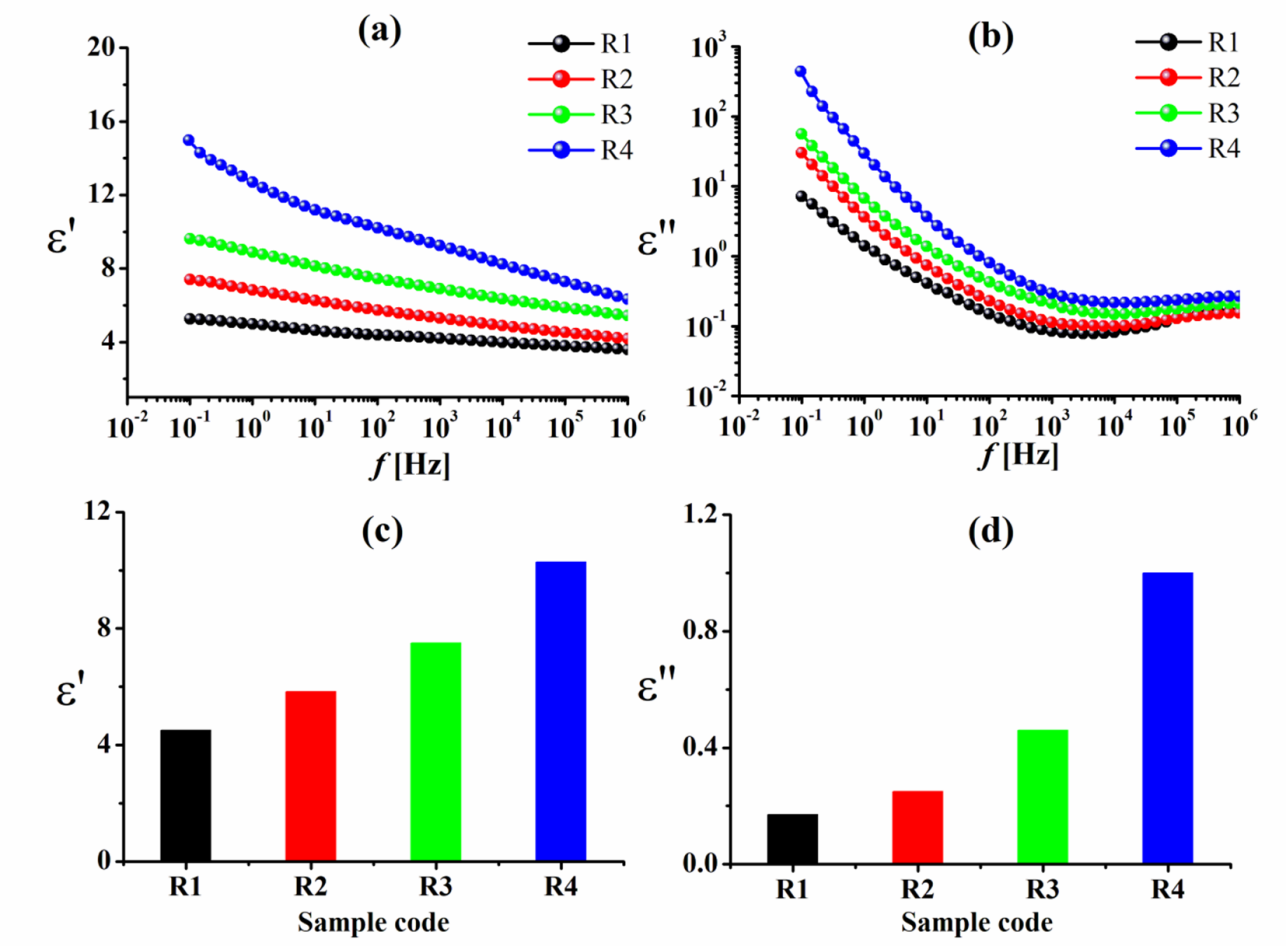
**a**, **b** The permittivity ε′ and the dielectric loss ε″ vs. frequency for rice straw pulp-based composites loaded with varying concentrations of Cu–LSF complex. **c**, **d** Dependence of the permittivity ε′ and the dielectric loss ε″ on Cu–LSF loading at fixed frequency (100 Hz).

The permittivity ε′ and dielectric loss ε″ of rice straw pulp-based composites, which were loaded with varying concentrations of Cu–LSF complex and immersed in FeCl_3_ solution, are shown in Fig. 7 at 30 °C, respectively. The permittivity ε′ shows saturation on the high-frequency side after dispersion in the low-frequency area for all samples^42^. It is also clear that as Cu–LSF complex concentration rose, so did the values of ε′ and ε″. The values of both ε′ and ε″ increase dramatically at low frequencies. Owing to the build-up of free charge at the interface between the electrode and the polymer film (electrode polarization effects), which obscures other relaxation processes, however, at greater Cu–LSF loadings, Cu–LSF particles tend to agglomerate, potentially reducing the interface interaction area between polymer chains and Cu–LSF particles. Such agglomeration could be minimized by the addition of anti-agglomeration agents^43^ or *via* complex surface treatment^44^.

The agglomerated Cu–LSF particles will act as vacancies between the polymer chains, enhancing their mobility. Furthermore, the presence of Cu may lead to increased localization of charge carriers, resulting in better ionic conductivity^15,45^. At a fixed, intermediate frequency of 100 Hz (see Fig. 7c, d), the addition of the Cu–LSF complex to the matrix certainly affected the values for both ε′ and ε″, as a result of the samples’ increased charge carrier density and corresponding rise in DC conductivity, respectively. It is also confirmed that the presence of non- Debye response is due to the broad ε″ *f* curves, which refer to the presence of more than one relaxation process.

**Fig. 8:**
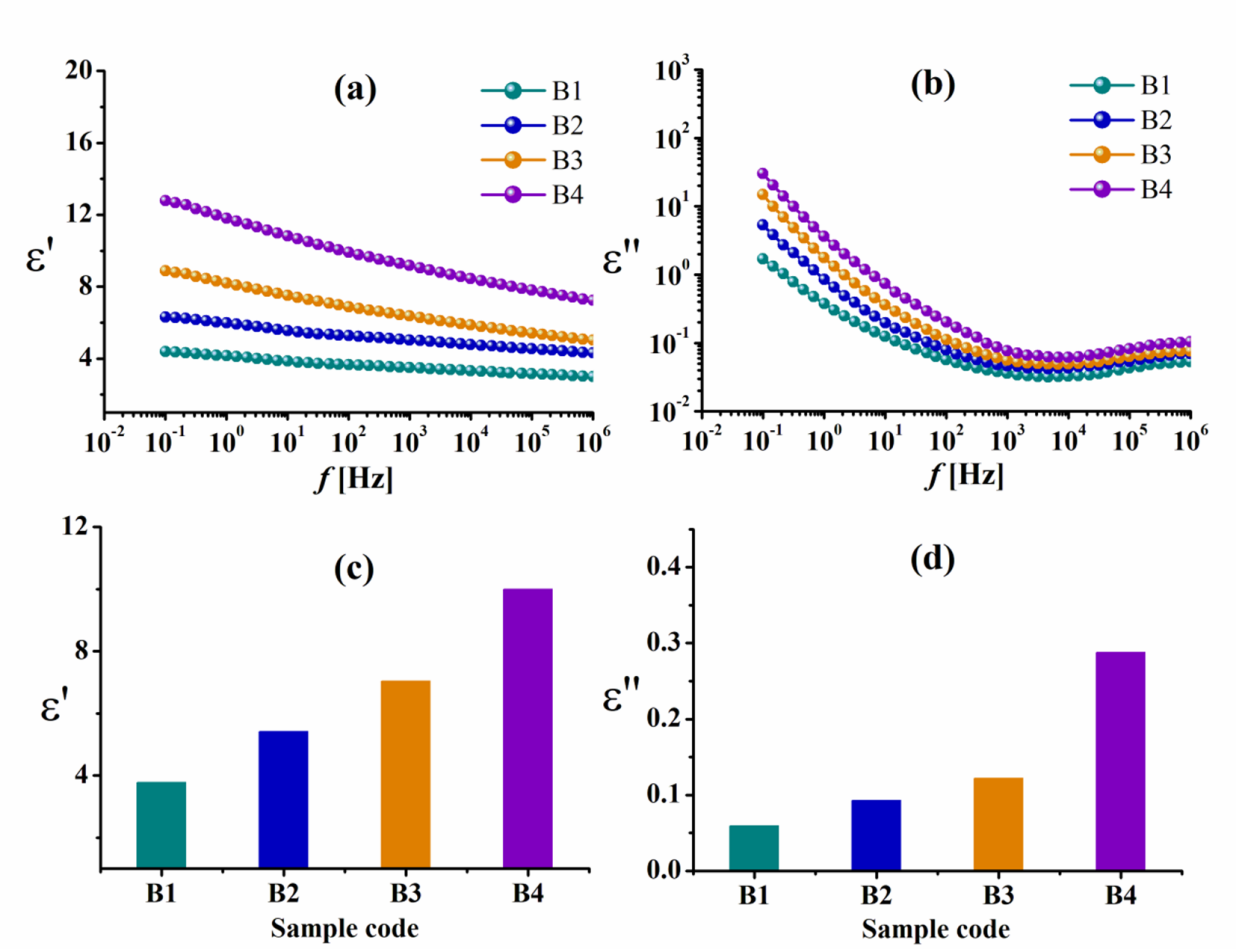
**a**, **b** The permittivity ε′ and the dielectric loss ε″ vs. frequency *f* for bagasse pulp-based blend loaded with varying concentrations of Cu–LSF complex. **c**, **d** Dependence of the permittivity ε′ and the dielectric loss ε″ on Cu loading at fixed frequency (100 Hz) in the presence of FeCl_3_.

Obviously, ε′ and ε″ of bagasse pulp-based composites, which were loaded with different concentrations of Cu–LSF complex and immersed in FeCl_3_ solution, are displayed in Fig. 8, measured at 30 °C. As the frequency of the applied AC field increases, ε′ and ε″ of bagasse-based pulp decrease. The improved polarization in the composite material is responsible for the permittivity’s rise with increased Cu–LSF complex loading. Conductive channels and polarizable particles are introduced into a polymer matrix by the addition of copper complex (Cu–LSF) and ferric chloride (FeCl_3_). As a result, the composite’s permittivity, or dielectric constant, rises.

By expanding the number of dipoles that can align with applied electric field, conductive fillers such as Cu–LSF complex and FeCl_3_ generally improve a polymer matrix’s capacity to store electrical energy. This dipole alignment increases the material’s permittivity by adding to its overall polarization, as shown in Fig. 8. Furthermore, when frequency increases, ε″, which is a measure of the material’s energy dissipation, also falls^46,47^.

However, it appears that the rice straw pulp-based composites exhibit a greater rise in permittivity with increasing Cu–LSF complex loading at a fixed frequency of 100 Hz (Fig. 7c, d) than the bagasse pulp-based composites (Fig. 8c, d). This pronounced rise in permittivity could be attributed to the higher content of insulating silica present in rice straw pulp^48^ as well as in the added Cu–LSF complex^25^.

**Fig. 9:**
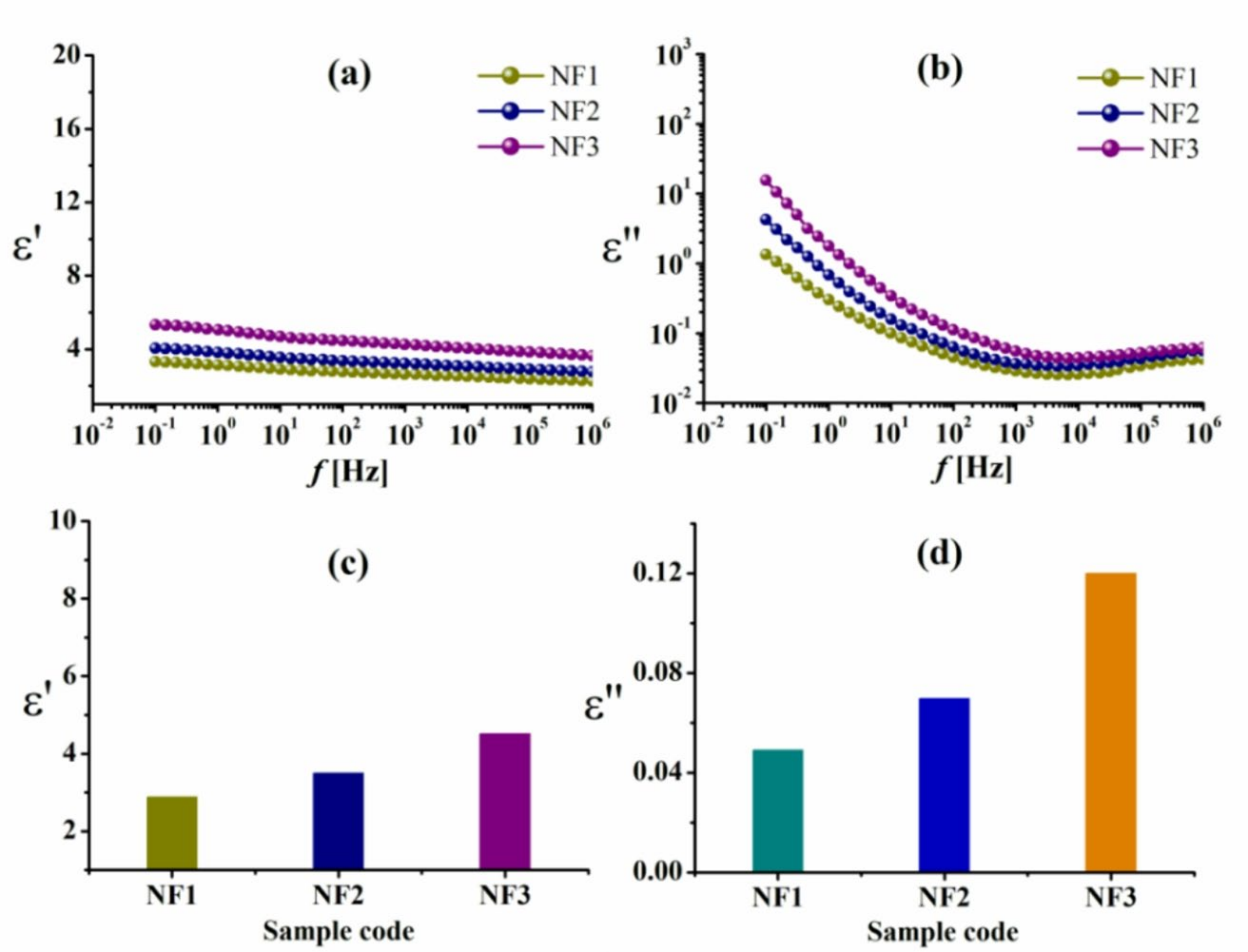
**a**, **b** The permittivity ε′ and the dielectric loss ε″ vs. frequency *f* for bagasse pulp-based blend loaded with varying concentrations of Cu–LSF complex without immersing in FeCl_3_ solution. **c**, **d** Dependence of the permittivity ε′ and the dielectric loss ε″ on Cu–LSF complex loading at fixed frequency (100 Hz).

Moreover, the values of ε′ and ε″ for the bagasse pulp/Cu–LSF complex in Fig. 9 without FeCl_3_ reduced compared to those immersed in FeCl_3_. It seems that doping FeCl_3_ in bagasse pulp/Cu–LSF complex influences its chemical and physical properties *via* enhancing cross-linkage^49^.

However, when we compare the data in Table 2 at a fixed frequency (10^6^ Hz) to the values of the same samples that were calculated at a fixed frequency of 100 Hz (which are shown in Figs. 7, 8 and 9), it is clear that the dielectric measurements in Table 2 are lower.

It can be concluded that these composites might be suitable for high-frequency applications, which was reached as a result of doing so. Because of its exceptionally low dielectric losses, which have the potential to lessen the amount of noise or attenuate the signals of high-frequency circuits, it is recommended for circuits that operate at frequencies more than one megahertz. However, bagasse pulp/Cu–FeCl_3_ composites exhibit high permittivity coupled with low losses, rendering them a good choice for application in energy storage devices, where moderate permittivity improves charge retention.

**Table 2:** The dielectric data at a fixed frequency (10^6^ Hz).

Sample code	Permittivity (ε′)	Loss tangent (tanδ) (tanδ = ε″/ ε′)	Dielectric loss (ε″)
Rice straw pulp/ Cu–FeCl_3_ composites			
R1	3.569	0.0436	0.156
R2	4.139	0.0372	0.154
R3	5.381	0.0390	0.209
R4	6.210	0.0437	0.271
Bagasse pulp/ Cu–FeCl_3_ composites			
B1	2.998	0.0178	0.053
B2	4.317	0.0161	0.069
B3	4.968	0.0152	0.076
B4	7.153	0.0146	0.104
Bagasse pulp/Cu composites			
NF1	2.267	0.0188	0.0426
NF2	2.763	0.0557	0.154
NF3	3.647	0.0168	0.0613

**Fig. 10:**
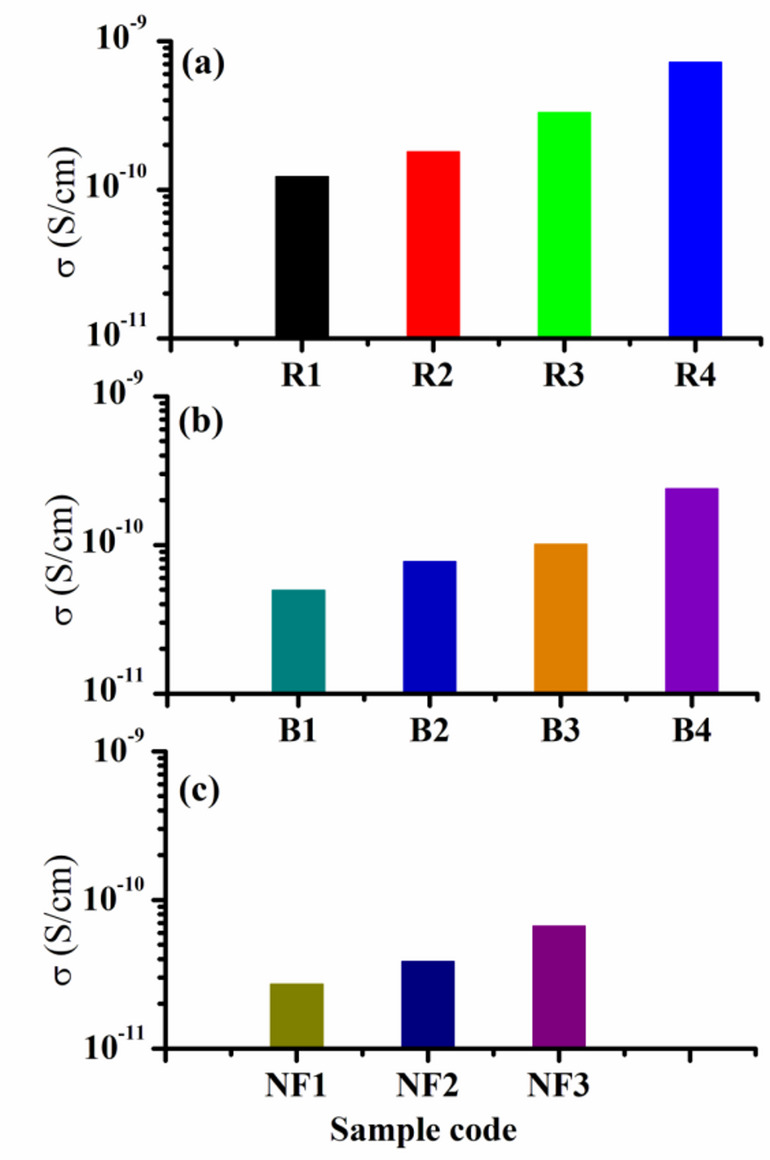
The dependence of the conductivity “σ” on Cu. **a**, **b** with FeCl_3_ and **c** without FeCl_3_.

The bagasse pulp-based composites with FeCl_3_ are expected to have an anticipated dispersion of Cu ions. As a result, during the preparation, hydrogen bonds with hydroxyl groups are formed, changing the chemical structure of the polymer repeating unit. This, in turn, increases the electrical conductivity (Fig. 10) by making the polymer chain more flexible and allowing better alignment and movement of charge carriers^50^.

Furthermore, these values increased from 1.52 × 10^−11^ S/cm for composites devoid of Cu–FeCl_3_ to 7.86 × 10^−10^ S/cm for composites including Cu–FeCl_3_ at ambient temperature (30 °C). These results of both composites strongly advocate for the use of composites with FeCl_3_ as antistatic materials. This renders it an optimal selection for safeguarding sensitive electronic components and packaging in electronics manufacturing against static damage. The appropriate conductivity range for materials used in antistatic applications is 10^−12^ to 10^−10^ S/cm^15,51^.

## Conclusion

Rice straw in Egypt is currently underutilized, often being discarded or incinerated after cultivation. The current study attempts to transform rice straw black liquor (RSBL) into a valuable material, aiming for clean pulp production and a cost-effective pulping process. Alkaline precipitation of the copper/silica complex (Cu–LSF hybrid) from RSBL affords effluent with low lignin, color, and COD contents ease to complete mineralization in the second treatment.

To end the pulping process with zero waste, we explore the utility of (Cu–LSF hybrid) in preparing new composites incorporating varying amounts of this conductive Cu–LSF hybrid into obtained rice straw cellulosic pulp. We then investigated their electrical characteristics, also comparing them to composites made from conventionally pulped bagasse.

Our results showed a direct relationship between the Cu–LSF complex concentration and the composite’s permittivity (ε′). This increase in permittivity could be attributed to enhanced polarization within the composite material. The addition of the copper complex and ferric chloride (FeCl_3_) introduces conductive channels and polarizable particles into the polymer matrix, boosting its ability to store electrical energy by increasing the number of dipoles that can align with an electric field. While higher Cu–LSF loadings generally improve permittivity, excessive amounts can lead to particle agglomeration, potentially reducing interaction with polymer chains and affecting the composite properties.

Notably, the rice straw-based composites demonstrated superior electrical properties compared to those made with bagasse pulp. This advantage is likely due to the higher percentage of dielectric silica present in the RSBL composites. Furthermore, doping pulp/Cu–LSF composites with FeCl_3_ positively influenced their chemical and physical properties by enhancing cross-linkage.

The strong electrical performance of both composite types makes them promising as antistatic materials and an optimal choice for safeguarding delicate electronic components and packaging in the electronics manufacturing industry. These findings support future exploration of sustainable, bio-based composites for antistatic packaging and flexible electronic devices.

## Data Availability

No datasets were generated or analysed during the current study.
